# Integrative Analysis of Transcriptome and GWAS Data to Identify the Hub Genes Associated With Milk Yield Trait in Buffalo

**DOI:** 10.3389/fgene.2019.00036

**Published:** 2019-02-05

**Authors:** Tingxian Deng, Aixin Liang, Shasha Liang, Xiaoya Ma, Xingrong Lu, Anqin Duan, Chunying Pang, Guohua Hua, Shenhe Liu, Giuseppe Campanile, Angela Salzano, Bianca Gasparrini, Gianluca Neglia, Xianwei Liang, Liguo Yang

**Affiliations:** ^1^Key Laboratory of Agricultural Animal Genetics, Breeding and Reproduction of Ministry of Education, Huazhong Agricultural University, Wuhan, China; ^2^Guangxi Provincial Key Laboratory of Buffalo Genetics, Breeding and Reproduction Technology, Buffalo Research Institute, Chinese Academy of Agricultural Sciences, Nanning, China; ^3^Department of Veterinary Medicine and Animal Productions, University of Naples Federico II, Naples, Italy

**Keywords:** buffalo, genome-wide association studies, hub genes, milk yield, RNA-seq, WGCNA

## Abstract

The mammary gland is the production organ in mammals that is of great importance for milk production and quality. However, characterization of the buffalo mammary gland transcriptome and identification of the valuable candidate genes that affect milk production is limited. Here, we performed the differential expressed genes (DEGs) analysis of mammary gland tissue on day 7, 50, 140, and 280 after calving and conducted gene-based genome-wide association studies (GWAS) of milk yield in 935 Mediterranean buffaloes. We then employed weighted gene co-expression network analysis (WGCNA) to identify specific modules and hub genes related to milk yield based on gene expression profiles and GWAS data. The results of the DEGs analysis showed that a total of 1,420 DEGs were detected across different lactation points. In the gene-based analysis, 976 genes were found to have genome-wide association (*P* ≤ 0.05) that could be defined as the nominally significant GWAS geneset (NSGG), 9 of which were suggestively associated with milk yield (*P* < 10^−4^). Using the WGCNA analysis, 544 and 225 genes associated with milk yield in the turquoise module were identified from DEGs and NSGG datasets, respectively. Several genes (including *BNIPL*, *TUBA1C*, *C2CD4B*, *DCP1B*, *MAP3K5*, *PDCD11*, *SRGAP1*, *GDPD5*, *BARX2*, *SCARA3*, *CTU2*, and *RPL27A*) were identified and considered as the hub genes because they were involved in multiple pathways related to milk production. Our findings provide an insight into the dynamic characterization of the buffalo mammary gland transcriptome, and these potential candidate genes may be valuable for future functional characterization of the buffalo mammary gland.

## Introduction

Water buffaloes (*Bubalus* *bubalis*) are known for their high-quality milk and meat consumption despite nutritional rations consisting of low-quality forage and fodder. Murrah, Nili-Ravi and Mediterranean breeds appear to be the top three dairy breeds of buffalo in the world with an average milk yield of 1800–2500, 1800–2400, and 2000–2800 liters in 300 days, respectively (Thomas, 2008; Medhammar et al., 2012). However, even the best milk producing buffalo breed cannot compare to Holstein cattle when it comes to average milk production. The lower milk production level may limit the development of dairy buffalo industry. Thus, the dairy buffalo industry prioritizes the improvement of milk production. Therefore, the implementation of new strategies, such as investigating the genetic architecture of milk yield, could facilitate a better understanding of different production traits, and these strategies may be used in high-yield buffalo breeding programs.

Transcriptome sequencing (RNA-Seq) is a sensitive, broad-spectrum detection tool for the analysis of gene expression pattern and provides insight into the mechanisms underlying complex traits in humans and livestock animals. The mammary gland is of great importance for milk production and quality. In recent years, numerous reports on the transcriptome profile analysis of the mammary gland have emerged in different livestock animals such as cattle (Cui et al., 2014), sheep (Suárez-Vega et al., 2016), and goat (Mobuchon et al., 2015). Moreover, different RNA sources including mammary gland biopsies (Dai et al., 2017), milk somatic cells (Salama et al., 2015) and milk fat globules (Cánovas et al., 2014) have been utilized to perform the mammary gland transcriptome analysis. However, RNA-Seq technology has not been used to describe the buffalo mammary gland transcriptome.

Genome-wide association studies (GWAS) are a powerful tool for investigating the genetic architecture of complex traits that have been widely used to identify the genetic variants and quantitative trait loci (QTL) affecting production traits among livestock (Jiang et al., 2010; Bush and Moore, 2012; Li et al., 2013). To date, traditional GWAS have had limited success in illustrating the genetic architecture underlying the complex traits because of a large number of loci with small effects (Visscher et al., 2012; Wood et al., 2014; Fang et al., 2017). The novel analytical methods to increase power should is necessary to overcome this limitation. The gene-based or pathway-based GWAS emerged as a novel approach that combines genetic information for all single nucleotide polymorphisms (SNPs) in a gene or pathway to increase the capability to find novel genes and generate more informative results (Neale and Sham, 2004; Wang et al., 2010; Xia et al., 2017). However, several issues complicate gene-based or pathway-based analysis such as variations in enrichment results across software tools, biased enrichment analyses due to concerned pathway membership, and the relatively unknown relationship between associated genes (Elbers et al., 2009; Farber, 2013). More importantly, information related to these issues is vital to understand how the associated genes influence the complex traits. This particular limitation may be improved by employing a weighted gene co-expression network analysis (WGCNA). The WGCNA algorithm can group genes into modules based on the gene co-expression similarities across the samples, resulting in a cluster of genes with similar functions (Langfelder and Horvath, 2008; Van-Nas et al., 2009; Liu et al., 2017). Therefore, WGCNA is a useful tool to identify the functional connections between genes in an unbiased manner using trait-relevant expression data.

In the present study, we performed the transcriptome analysis of lactating mammary gland tissue at different lactation stages of buffaloes. WGCNA was then used to investigate the differentially expressed genes (DEGs) associated with milk yield. Previously published GWAS datasets were used to conduct the system-level study of milk yield traits by WGCNA. Finally, we integrated the analyses of the two datasets to identify hub genes affecting milk production. As a result, an exclusive set of genes affecting milk production were identified. These genes may play a vital role in mammary gland development and lactation.

## Materials and Methods

### Mammary Gland Tissue

All experimental designs and animal care protocols were approved by the Animal Ethics Committee of the Buffalo Research Institute, Chinese Academy of Agricultural Sciences (BRI-CAAS) and Huazhong Agricultural University.

A total of eight Murrah buffaloes were selected and used for the biopsy at the BRI-CAAS. Lactation history (including milk yield and parity) was used for sample selection. The selected animals were in their 3rd and 4th parity. Mammary gland tissue samples were collected on day 7 (D7), 50 (D50), 140 (D140), and 280 (D280) after calving and immediately preserved in liquid nitrogen until use. Two replicates were used for each lactation point in this experiment. The sampling points represented the different physiological stages of the mammary gland across lactation. D7 represented early lactation, D50 corresponded peak lactation, and D140 and D280 represented the mid and late stages of lactation.

### RNA Extraction, cDNA Library Construction and RNA Sequencing

Total RNA from approximately 3 ∼ 5 mg of mammary gland biopsies was isolated using Trizol reagent according to the manufacturer’s protocol (Invitrogen, Carlsbad, CA, United States). The total RNA was quantified and evaluated using the Agilent Bioanalyzer 2100 Instrument (Agilent Technologies, Beijing, China) with the RNA Integrity Number (RIN) value.

The cDNA library for each sample was prepared by Illumina TruSeq^TM^ RNA Sample Preparation Kit (Illumina, San Diego, CA, United States) following the manufacturer’s instructions. Briefly, 5 μg of RNA for each sample was used for RNA-seq library preparation. The poly (A) mRNAs were isolated from total RNA using the Oligo (dT) magnetic beads. The cDNA synthesis was performed using the SuperScript II, DNA Polymerase I and RNase H treatment, and then chemically fragmented to ∼200 nt fragments and enriched with PCR to create the final cDNA libraries. Eight cDNA libraries were sequenced on the Illumina HiSeq^TM^ 2000 platform (Illumina, San Diego, CA, United States). The sequencing data were deposited in the NCBI SRA database (BioProject ID: PRJNA480718). Supplementary Figure S1 describes the main steps and bioinformatics used for data analysis.

### Differential Gene Expression Analysis

The sequencing quality of raw fastaq files was checked using the FastQC version 0.11.7 software^1^. After quality control, the clean data were used to align reads to the buffalo genome (*UOA_WB_1*) allowing 2 bp mismatch using the TopHat version 2.1.1 software (Kim et al., 2013). The expression level of each transcript was represented by the Trimmed Mean of *M*-values (TMM) described in and implemented in the edgeR R-package (Robinson et al., 2010). The differential analysis for pairwise contrasts was performed using the DESeq2 R-package (Love et al., 2014). The Benjamini and Hochberg corrected *P*-value ≤ 0.05 and FoldChange > 2 were defined as the selection threshold for the DEGs.

### Gene-Based Association Analysis

Two previously published GWAS datasets (Iamartino et al., 2017; Liu et al., 2018) were used to perform the gene-based association analysis aiming to investigate the candidate genes associated with milk yield in buffaloes. All SNPs in the GWAS dataset were converted to gene lists using MAGMA (de Leeuw et al., 2015) software. Prior to MAGMA analysis, a total of 33,478 buffalo genes (*UOA_WB_1*) were extracted from the buffalo GTF annotation file using the in-house scripts. Because it was previously reported that approximately 90% of SNPs affecting eQTL were observed within 15 kb from the 5′ and 3′-end of a gene (Pickrell et al., 2010), a window of 7.5 Kb upstream and 7.5 Kb downstream of the gene was selected for SNP mapping in the present study. We performed the gene-level association analysis with the multi = all command, which computed all three models: (1) principal components regression model, (2) SNP-wise Mean (snp-wise = mean) model, and (3) SNP-wise Top 1 (snp-wise = top) model for each gene. The three *P*-values were combined into an aggregate *P*-value. According to the currently published buffalo GWAS reports (De Camargo et al., 2015; El-Halawany et al., 2017; Iamartino et al., 2017; Liu et al., 2018), the 10^−4^ was set as the threshold of gene-based analysis in this work. The genes with a joint *P* < 0.05 for milk yield were defined as the nominally significant GWAS gene set (NSGG) for further analysis.

### Co-expression Network Analysis for the DEGs and NSGG

For the gene expression matrix from the DEGs and NSGG, we employed the WGCNA R-package (Langfelder and Horvath, 2008) to conduct the co-expression network construction analysis. Briefly, the gradient method was used to explore the value of the adjacency matrix weight parameter: power (values ranged from 1 to 30). Then, the topological overlap matrix (TOM) was constructed using correlation expression values and used for the hierarchical clustering analysis. The gene module was detected using a dynamic tree cutting algorithm. In this study, powers with 17 and 9 were set as the power cutoff for the DEGs and NSGG dataset, respectively. Because these power values were chosen based on the scale-free topology criterion (Zhang and Horvath, 2005), resulting in a scale-free topology index (R2) of 0.85. For both datasets, gene modules were constructed using the dynamic hierarchical tree-cut algorithm with the following parameters: minModuleSize = 40, deepSplit = 2, corFnc = “bicor,” mergeCutHeight = 0.25, and networkType = “signed hybrid.” The co-expression module structure was visualized by the gene dendrogram plots.

### Identification of Modules Associated With Milk Yield

To identify modules that were significantly associated with milk yield trait, the resulting module genes were used to calculate the module eigengenes (MEs, or the first principal component of the module). In general, the expression patterns of all genes could be summarized into a single characteristic expression profile within a given module (Yuan et al., 2017). Therefore, the correlation analysis between MEs and milk yield was conducted to identify the relevant module.

### Hub Gene Analysis

Hub gene is usually used as an abbreviation of a highly connected gene that tends to have high connectivity in the co-expression modules. The module membership (MM) was defined as the correlation between each gene’s expression and its MEs. The gene significance (GS) was defined as each gene’s correlation with traits of interest. Genes with the highest MM and highest GS in modules of interest were set as the candidates for further analysis (Fuller et al., 2007). In this study, for the DEGs and NSGG modules, the intramodular hub genes that were associated with milk yield were chosen by the following threshold: GS > 0.2, MM > 0.8, and *P* ≤ 0.05. The common hub genes both in co-expression network and NSGG network were regarded as “real” hub genes for further analysis. Gene interaction network of the common hub genes was visualized using the Cytoscape ver3.6 software (Shannon et al., 2003).

### Functional Annotation and Enrichment Analysis

The gene ontology (GO) analysis and Kyoto Encyclopedia of Genes and Genomes (KEGG) pathway analysis for the DEGs, module genes and hub genes were performed using the KEGG Orthology-Based Annotation System (KOBAS) 3.0 (Xie et al., 2011) with a cutoff of *P* < 0.05. The plot results were visualized using the in-house R scripts.

### Real-Time RT-PCR Confirmation of Hub Genes

Total RNA was used for the cDNA synthesis using the RevertAid First Strand cDNA Synthesis kit (Thermo Fisher Scientific, Waltham, MA, United States). The quantitative real-time PCR (qPCR) was conducted using the LightCycler 480 (Roche, CH), and each reaction was performed in triplicate. Relative mRNA expression levels were calculated using the 2^−^ΔΔCt method (Livak and Schmittgen, 2001) and normalized using the *GAPDH* expression analysis. All the primers for the qPCR are shown in Supplementary Table S1.

## Results

### mRNA Expression Profiles in the Lactating Mammary Gland Tissues

In order to investigate the global mRNA expression changes in the lactating mammary gland tissues, we performed the high-throughput profiling analysis of eight mammary gland tissues at four different lactation points (D7, D50, D140, and D280). After filtering, approximately 22.14 Mb clean reads per sample were generated. Approximately 90.89% of the clean reads for each sample were mapped to the buffalo genome (*UOA_WB_1*), and 74.79% of clean reads were mapped to unique reads (Supplementary Table S2). Moreover, the Pearson correlation coefficients (PCCs) analysis between all RNA-seq samples showed that the PCCs between samples were ranged from 0.9318 to 1.0000 (Supplementary Figure S2), suggesting that our RNA-seq results met the requirement of following DEG identification.

**FIGURE 1:**
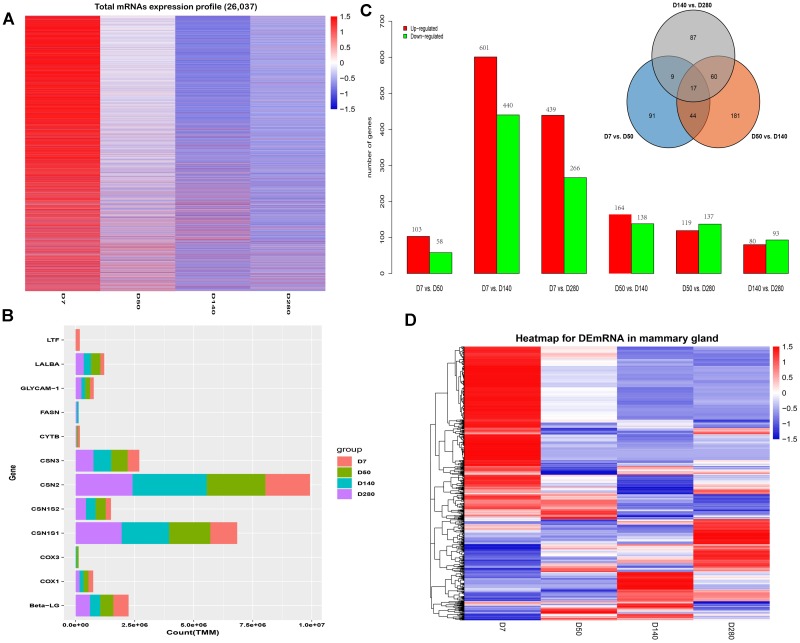
Expression profiles of buffalo mammary gland tissues during four lactation point. **(A)** The cluster heat map of all mRNAs expression at different stages during lactation. **(B)** Bar plots are showing top10 genes with highest TPM values across the four points. **(C)** Bar plots showing differentially expressed genes by the pairwise comparison. **(D)** Hierarchical clustering is showing often up and down-regulated mRNAs across the four points.

Herein, a total of 26,037 mRNAs were detected, and their expression profiles are shown in Figure 1A. Similarly, the genes with the highest TMM values in mammary gland tissues at D7, D50, D140, and D280 during lactation were identified (Figure 1B). Six comparison groups (D7 vs. D50, D7 vs. D140, D7 vs. D280, D50 vs. D140, D50 vs. D280, and D140 vs. D280) were set according to the lactation points, and the results are presented in Figure 1C. A total of 103, 601, and 439 DEGs were up-regulated in the D50, D140, and D280 groups compared to the D7 group. In contrast, a total of 58, 440, and 266 DEGs were down-regulated in the D50, D140, and D280 groups. Compared with those in D50, a total of 164 and 119 DEGs were found to be up-regulated in D140 and D280, respectively, whereas a total of 138 and 137 down-regulated mRNAs were discovered in D140 and D280. In the D140 vs. D280 group, we found 80 up- and 93 down-regulated DEGs. The unified set of DEGs containing a total of 1,420 mRNAs was found among all comparison groups. Notably, a total of 17 genes were shared among the different lactation points, and 91, 181, and 87 unique genes were found in the D7 vs. D50, D50 vs. D140, and D140 vs. D280 groups, respectively (Figure 1C). Hierarchical cluster analysis of the unique DEGs among all comparison groups was performed to examine expression patterns (Figure 1D).

### Gene-Based Association Analysis

**FIGURE 2:**
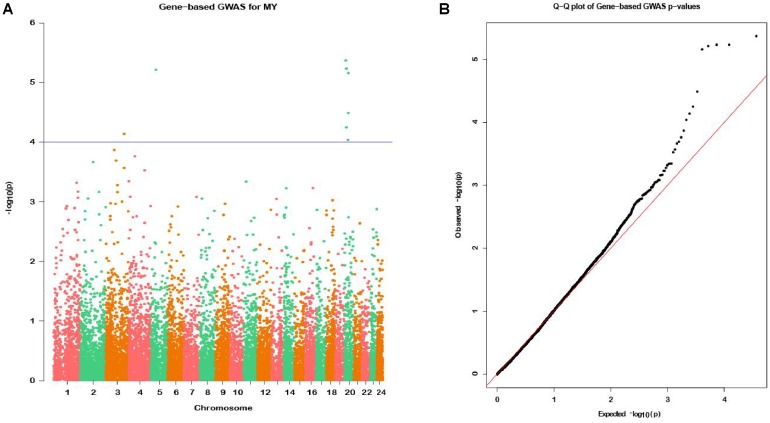
Manhattan plot **(A)** of -log10 (*P*-values) and Quantile-Quantile plot **(B)** of *P*-values for milk yield from the gene-based method. The blue horizontal line indicates the suggestive significance level [-log10(1e-4)].

In the gene-based analysis, the suggestively significant genes affecting milk yield are shown in Figure 2A. The corresponding genes are listed in Table 1, including their starting and ending positions in the genes, ensemble IDs, buffalo Autosome, Gene symbol, and the numbers of the mapping SNPs. A total of nine genes (*TCL1B*, *PDIA3*, *LOC112581059*, *COCH*, *STRN3*, *CCDC88C*, *NPAS3*, *NUDT2*, and *UBE4B*) were discovered. The Q-Q plot of milk yield in the gene-based analysis demonstrated that there was no inflation or systematic bias (Figure 2B). Therefore, a total of 976 genes with *P* ≤ 0.05 were regarded as the NSGG for further analysis in this study (Supplementary Table S3).

**Table 1:** Significant genes identified for milk yield by gene-based GWAS method.

GENE	CHR	Start (bp)	Stop (bp)	NSNPS	*P*-value	Symbol
112580920	20	8966541	8986082	1	4.26E–06	TCL1B
102408568	20	14035135	14072539	1	5.84E–06	PDIA3
112581059	20	14050439	14065548	1	5.84E–06	LOC112581059
102394136	20	28040397	28069174	1	6.94E–06	COCH
102396060	20	27932196	28053180	3	3.25E–05	STRN3
102411925	20	13250379	13408926	5	5.64E–05	CCDC88C
102402688	20	25285048	26264479	19	9.14E–05	NPAS3
102400876	3	1.38E + 08	1.38E + 08	1	7.27E–05	NUDT2
102409055	5	37551819	37688935	4	6.12E–06	UBE4B

*GENE, gene ID name in buffalo genome; CHR, *buffalo* Autosome; Start (bp), the start position (bp) of genes; Stop (bp), the end position (bp) of genes; NSNPs, numbers of SNPs mapped to a gene. Symbol: the corresponding gene name.*

### Network Analysis of DEGs and NSGG

**FIGURE 3:**
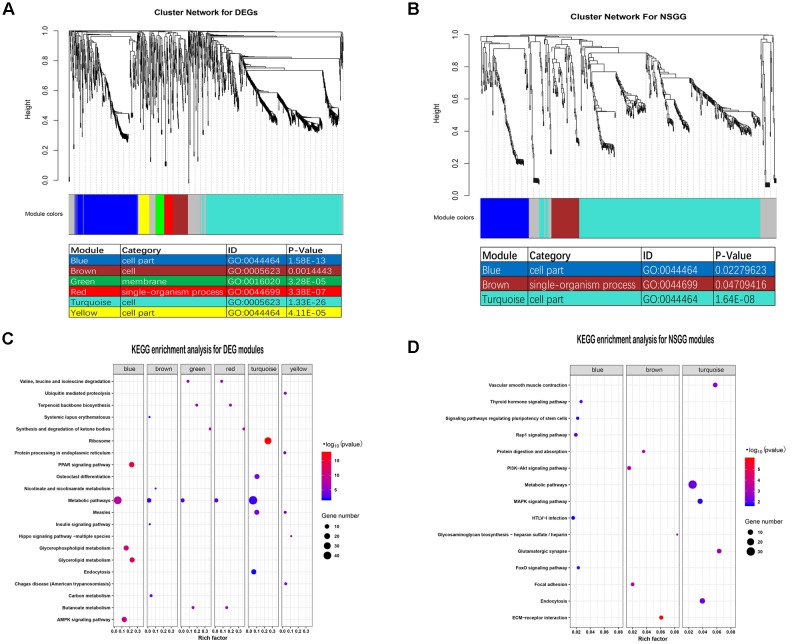
Identification of modules and functional annotation analysis for the module genes. **(A)** Module detection for the DEGs dataset and GO analysis for the module genes. **(B)** Module detection for the NSGG dataset and GO analysis for the module genes. **(C)** KEGG enrichment analysis for module genes from the DEGs dataset. **(D)** KEGG enrichment analysis for module genes from the NSGG dataset.

To evaluate the co-expression relationships for the DEGs and NSGG datasets, we used the WGCNA algorithm to define trends in gene co-expression across mammary gland tissues at different lactation stages. For the DEGs, a total of seven co-expression modules were detected (Figure 3A), with 164 DEGs failing to cluster into a distinct group (known as the gray module). The size of modules ranged from 44 (red module) to 715 DEGs (turquoise module). Most of these modules were significantly enriched in mammary gland-specific gene ontologies as well as established cellular functions (Figure 3A). These modules presented the strongest correlation with categories of the cell (turquoise module), cell part (blue module), cell (brown module), cell part (yellow module), single-organism process (red module), and membrane (green module). Interestingly, the metabolic pathways were found to be significantly enriched (Top 1) in all identified DEGs modules except for the yellow module (Figure 3C).

For the NSGG, a total of 402 genes were assigned into the turquoise module (Figure 3B), followed by the blue module (103 genes) and brown module (59 genes), and the 65 uncorrelated genes were grouped into a gray module. The turquoise and blue module genes both were significantly enriched in the cell part term, while the brown module genes were significantly enriched in the single-organism process term. As shown in Figure 3D, the genes in the turquoise module were found to be significantly enriched in the following top3 pathways including the metabolic pathways, endocytosis, and MAPK signaling pathway. The blue and brown module genes were significantly enriched in the Rap1 signaling pathway and ECM-receptor interaction, respectively.

### Identification of Modules Associated With Milk Yield

**FIGURE 4:**
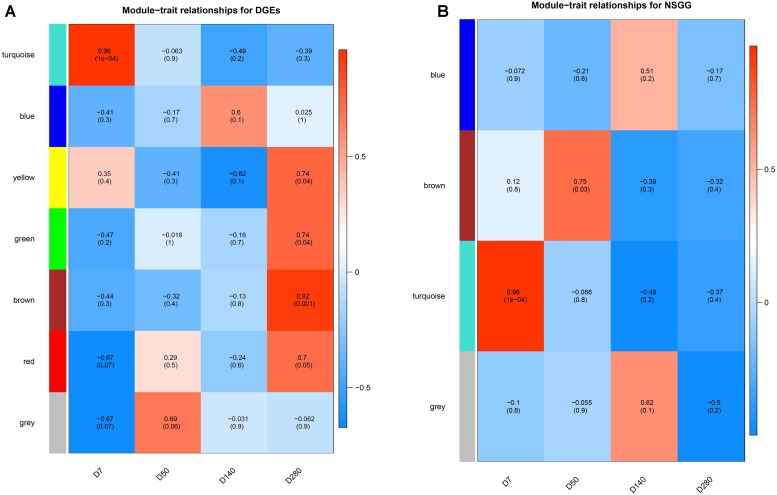
Heatmap of the correlation between module eigengenes and milk yield. **(A)** module-traits analysis for the DEGs dataset. **(B)** module-traits analysis for the NSGG dataset.

To understand the module-trait significance, we correlated the selected modules with milk production and searched for significant associations. A total of five modules (turquoise, yellow, green, brown, and red) of DEGs were found to have a significantly positive correlation with milk yield at D7 and D280, respectively (Figure 4A). DEGs clustered in the turquoise module (*n* = 715) had the strongest positive correlation at D7 (*r* = 0.96, *P* = 1e-04), while the brown module displayed the highest positive correlation at D280 (*r* = 0.92, *P* = 0.001). For the NSGG modules, genes assigned in the turquoise module (*n* = 402) exhibited the strongest positive correlation with milk yield at D7 (*r* = 0.96, *P* = 1e-04), while the brown module genes displayed a significantly positive correlation with milk yield at D50 (*r* = 0.75, *P* = 0.03) (Figure 4B). The correction analysis of the DEGs and NSGG indicated that the turquoise module exhibited a higher ability to indicate external traits accurately than other modules. Moreover, our GO and KEGG analysis showed that the turquoise module genes were significantly enriched in the metabolic pathways, suggesting that these genes might be related to mammary gland development and secretion.

### Hub Genes Analysis

**FIGURE 5:**
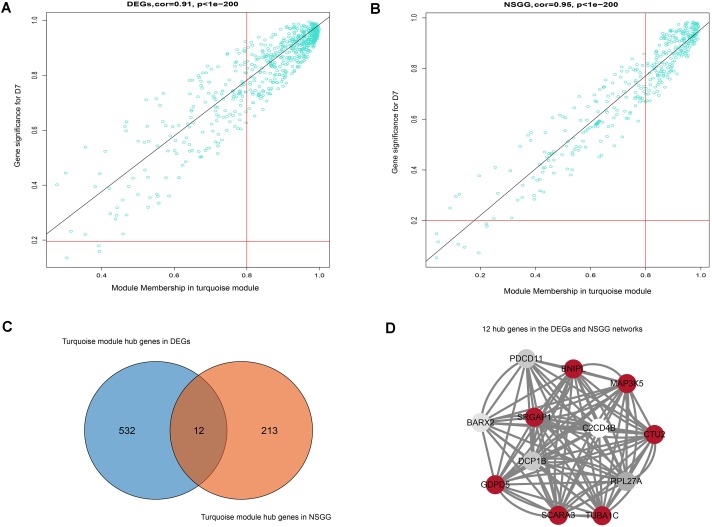
Hub genes detection and network construction analysis. **(A)** Scatter plot of module eigengenes in the turquoise module from DEGs dataset. **(B)** Scatter plot of module eigengenes in the turquoise module from NSGG dataset. **(C)** The Venn diagram of the DEGs and NSGG hub genes. **(D)** Hub gene interaction network of in the turquoise module from the DEGs and NSGG dataset. The color intensity in each node was proportional to the TOM values calculated by WGCNA (the higher TOM values were in a circle with red, whereas the lower TOM values were in a circle with white).

To further identify the hub genes in the turquoise module that were associated with milk yield, we investigated MM and GS values of genes and set the genes with the highest values of MM and GS in modules of interest as the hub genes. Figure 5A,B shows the scatter plots of GS related to milk yield at D7 versus MM in the turquoise module. A total of 544 and 225 genes highly related to milk yield in the turquoise module were identified and set as hub genes of the DEGs and NSGG, respectively. Interestingly, a total of 12 genes (*BNIPL*, *TUBA1C*, *C2CD4B*, *DCP1B*, *MAP3K5*, *PDCD11*, *SRGAP1*, *GDPD5*, *BARX2*, *SCARA3*, *CTU2*, and *RPL27A*) were found to be shared by two modules and were named “real” hub genes (Figure 5C and Table 2). The visualized plot of the gene-gene interaction network for the 12 hub genes was shown in Figure 5D. The GO analysis found most of the genes were enriched in the intracellular organelle, followed by the organelle, intracellular part, intracellular and cell part (Table 3). Notably, the *MAP3K5* and *TUBA1C* genes were significantly enriched in the apoptosis pathway (Table 3; *P* < 0.05). We further validated the expression level of the 12 hub genes using qPCR, showing that a similar tendency was identified between the mRNA expression level from the RNA-Seq and qPCR (Supplementary Figure S3).

**Table 2:** List of the overlapping hub genes in the turquoise module from the DEGs and NSGG.

Gene	Full name	Reference
*BNIPL*	BCL2/adenovirus E1B 19 kDa protein-interacting protein 2	/
*TUBA1C*	Tubulin alpha-1C chain-like	/
*C2CD4B*	C2 calcium-dependent domain-containing protein 4A	/
*DCP1B*	mRNA-decapping enzyme 1B	/
*MAP3K5*	Mitogen-activated protein kinase kinase kinase 5	Do et al., 2017
*PDCD11*	Protein RRP5 homolog	/
*SRGAP1*	SLIT-ROBO Rho GTPase-activating protein 1	/
*GDPD5*	Glycerophosphodiester phosphodiesterase domain-containing protein 5	/
*BARX2*	Homeobox protein BarH-like 2	/
*SCARA3*	Scavenger receptor class A member 3 isoform X1	/
*CTU2*	Cytoplasmic tRNA 2-thiolation protein 2	/
*RPL27A*	60S ribosomal protein L27a	Yamada et al., 2009

**Table 3:** The results of GO and pathway enrichment analysis for the overlapping hub genes clustered in the turquoise module.

Category	Term	ID	*P*-value	Gene symbol
GO	Intracellular organelle	GO:0043229	0.001418	*C2CD4B, RPL27A, BNIPL, PDCD11, DCP1B, GDPD5, TUBA1C, CTU2*
	Organelle	GO:0043226	0.002491	*C2CD4B, RPL27A, BNIPL, PDCD11, DCP1B, GDPD5, TUBA1C, CTU2*
	Intracellular part	GO:0044424	0.00389	*C2CD4B, RPL27A, BNIPL, PDCD11, DCP1B, GDPD5, TUBA1C, CTU2*
	Intracellular	GO:0005622	0.004957	*C2CD4B, RPL27A, BNIPL, PDCD11, DCP1B, GDPD5, TUBA1C, CTU2*
	Cell part	GO:0044464	0.012614	*C2CD4B, RPL27A, BNIPL, PDCD11, DCP1B, GDPD5, TUBA1C, CTU2*
Pathways	apoptosis	bta04210	0.001751	*MAP3K5, TUBA1C*

## Discussion

Transcriptome analysis to reveal genes actively expressed in specific tissues and species of interest is particularly useful to non-model organisms whose full genome data are not available yet (Waiho et al., 2017). To date, there have been numerous studies of global gene expression alterations in the mammary gland (Bionaz and Loor, 2007; Shi et al., 2015; Suárez-Vega et al., 2016). These studies have indicated temporal and spatial specificity in the transcriptome profiles of the mammary gland in different species. In the present study, eight libraries of mammary gland tissues corresponding to the different days of lactation (D7, D50, D140, and D280) were constructed and used for high-throughput sequencing. A total of 26,037 mRNAs were detected across the samples, and several genes with the highest TMM values at different lactation points were identified, such as the *CSN1S2* (casein-α-S2), *CSN1S1* (α-S1-casein), *CSN2* (β-casein), *CSN3* (κ-casein), *Beta-LG* (β-lactoglobulin), *GLYCAM1* (Glycosylation-dependent cell adhesion molecule 1), and *LALBA* (α-lactoalbumin). All of these genes except *GLYCAM1* have been reported to be highly expressed in mammary cells in other species such as cattle (Suárez-Vega et al., 2015) and human (Lemay et al., 2013), reflecting the similar biological function of the lactating mammary gland.

Identifying DEGs is critical to any transcriptome analysis. This study found that most of the variations in the expression of DEGs were identified in D7 vs. D140 group, followed by D7 vs. D280, D50 vs. D140, D50 vs. D280, D140 vs. D280, and D7 vs. D50 groups. This trend is consistent with the results reported by Bionaz et al. (2012). Importantly, a unified set of DEGs containing 1,420 mRNAs was identified among all comparison groups, suggesting these DEGs may have temporal specificity and reflect the physiological activity of the lactating mammary gland. Interestingly, these DEGs assigned into 6 co-expression modules using WGCNA analysis. In particular, the turquoise module had a significantly positive correlation with milk yield at D7, and four modules (green, brown, red, and yellow) were found to be associated with milk yield at D280. The GO analysis showed that most of these modules were significantly enriched for mammary gland-specific GO as well as established cellular functions. Early lactation is known to be a critical period for mammary development, in which the mammary wet weight and total DNA content continue to increase (Akersr et al., 1981), resulting in the increased milk production in some species. Our data showed that the turquoise module genes had the strongest correlation with the metabolic pathways, ribosome, and endocytosis, suggesting these genes might be involved in the metabolism, protein process, and transport in the mammary epithelial cell. During late lactation, the animals prioritized increasing body tissue over milk production under thermal stress (Kim et al., 2010). Our data revealed that the four (brown, green, red, and yellow) module genes were significantly enriched in the metabolic pathways and Chagas disease (American trypanosomiasis), suggesting that these genes might be involved in energy metabolism.

Genome-Wide Association Studies is another effective method for mining the candidate genes responsible for the traits of interest, although these loci reveal only a small proportion of the genetic risk of the complex traits. Using the gene-level GWAS analysis, we identified a total of 9 genes (*TCL1B*, *PDIA3*, *LOC112581059*, *COCH*, *STRN3*, *CCDC88C*, *NPAS3*, *NUDT2*, and *UBE4B*) associated with milk yield. Some genes including the *PDIA3* (Janjanam et al., 2014), *CCDC88C* (Jiang et al., 2018), and *UBE4B* (Wickramasinghe et al., 2012) have been reported to be associated with milk production. Laine et al. (2002) reported that *TCL1B* is one member of the TCL1 proto-oncogene family that could enhance the phosphorylation and activation of AKT1 and AKT2, which is involved in the PI3K-Akt signaling pathway related to mammary gland development and lactation (Korkaya et al., 2009). Oka et al. (2011) highlighted that the *NUDT2* gene could asymmetrically hydrolyze Ap4A and regulate the intracellular Ap4A level that is associated with a wide variety of basic cellular functions including protein synthesis, cell contact growth inhibition and apoptosis. This association indicates that this gene might be involved in the functional regulation in the mammary epithelium cell. Moreover, previous studies reported that a potential limitation of both the single-marker and the gene-based GWAS is that these two types of GWAS do not provide functional information of associated genes (Farber, 2013; Xia et al., 2017). To overcome the limitation, we further performed a WGCNA analysis to investigate the functional connections between genes in the NSGG dataset. Interestingly, we found that a total of 402 genes were assigned into the turquoise module and 65 uncorrelated genes were grouped into a gray module. Notably, the turquoise module genes (63.91%) associated with milk yield at D7, as most of them were significantly enriched in the metabolic pathways. Our results suggest that these genes might be involved in the biological regulation of mammary gland development and early lactation.

One of the contributions of this study is that hub genes were identified by using the intramodular connectivity of genes in modules. The data show that the hub genes with the highest MM and GS in modules of interest should be considered as the natural candidates for further research (Ghazalpour et al., 2006; Horvath et al., 2006; Oldham et al., 2006). This study identified turquoise module genes associated with milk yield at D7 in the DEGs and NSGG datasets. Based on these findings, a total of 544 and 225 genes were separately considered as the hub genes from the DEGs and NSGG datasets. Notably, a total of 12 overlapping genes (*BNIPL*, *TUBA1C*, *C2CD4B*, *DCP1B*, *MAP3K5*, *PDCD11*, *SRGAP1*, *GDPD5*, *BARX2*, *SCARA3*, *CTU2*, and *RPL27A*) were found within the DEGs and NSGG networks and may be regarded as “real” hub genes. The proportion of common hub genes in the NSGG turquoise module (5.33%) was higher than that of the DEGs turquoise module (2.21%). Functional annotation analysis showed that many of the shared genes (*C2CD4B, RPL27A, BNIPL, PDCD11, DCP1B, GDPD5, TUBA1C*, and *CTU2*) were significantly enriched in the intracellular organelle, followed by the organelle, intracellular part, intracellular and cell part. For example, Cao et al. (2016) reported the GDPD5 silencing could decrease the cell proliferation, migration, and invasion of breast cancer. In addition, it should be noted that two genes (*MAP3K5* and *TUBA1C*) were significantly enriched in the apoptosis pathway. This pathway has been demonstrated to be involved in mammary gland development and lactation (Lund et al., 1996; Green and Streuli, 2004; Watson, 2006). In fact, *MAP3K5* (known as ASK1) is a member of the MAP3K family and has been identified as the upstream activator of Jun N-terminal kinases (JNKs) and p38 MAPKs pathways involved in mammary gland development (Wagner and Nebreda, 2009; Whyte et al., 2009; Cellurale et al., 2012). Do et al. (2017) reported that MAP3K5 gene had a significant genetic effect on lactation persistency in Canadian Holstein cattle. Moreover, *TUBA1C* is a member of the tubulin family that plays a vital role in the maintenance of cell morphology, movement, and intracellular transport (Hammond et al., 2008; Zhao et al., 2014). Loizzi and Shao (1990) reported that tubulin could increase with lactation in rat and pig mammary alveolar cells. These results suggest that the *TUBA1C* gene may be involved in many cell processes in the mammary gland, thereby affecting mammary gland development and lactation. Consequently, it can be assumed that these genes serve as the candidate genes affecting the mammary gland development and lactation. However, these findings remain to be confirmed.

In summary, this study is the first attempt to report the transcriptome profiles of the lactating mammary gland at different stages in dairy buffalo and identify the turquoise module genes associated with milk yield using the WGCNA algorithm. Twelve hub genes associated with milk yield were identified through a combination of transcriptome and GWAS data, two of which were predicted to be significantly enriched in the apoptosis pathway. Our findings provide an insight into the dynamic characterization of buffalo mammary gland transcriptome, and the identified candidate genes provide valuable information for future functional characterization. This study contributes to a better understanding of the genetic mechanisms underlying the milk production trait in buffaloes.

## Data Availability Statement

The genotype and phenotype datasets of this study are provided as the Supplementary Data Sheets S1, S2.

## Author Contributions

TD, XM, CP, GC, AS, BG, GN, and AL collected the buffalo blood and mammary gland tissue samples. SSL, XRL, AD, and SHL isolated genomic DNA and RNA. TD created and carried out the analysis, interpreted the data and wrote the manuscript. TD, XWL, and LY developed the study and participated in its design and coordination. LY, GH, and TD reviewed the manuscript. All authors have read and approved the manuscript.

## Conflict of Interest Statement

The authors declare that the research was conducted in the absence of any commercial or financial relationships that could be construed as a potential conflict of interest.

## Footnotes

1 http://www.bioinformatics.babraham.ac.uk/projects/fastqc/

## Supplementary Material

FIGURE S1 The main steps and bioinformatics used for data analysis in the present study.

FIGURE S2 The PCA analysis of the studied samples in the present study.

FIGURE S3 Validation of the expression of the 12 hub genes by qPCR. Gene expression levels were determined by qPCR and are presented as mean±SD values calculated by the 2^ΔCt^ method.

TABLE S1 Primer list of RT-qPCR.

TABLE S2 Describes of sequencing data for the eight mammary gland tissues.

TABLE S3 List of the NSGG using the gene-based association analysis.

DATA SHEET S1 The genotype datasets of this study.

DATA SHEET S2 The phenotype datasets of this study.
