# Supplementary figures and images for: Integrative Analysis of Transcriptome and GWAS Data to Identify the Hub Genes Associated With Milk Yield Trait in Buffalo
Source: Front Genet. 2019 Feb 5;10:36. doi: 10.3389/fgene.2019.00036 (PMC6371051; doi:10.3389/fgene.2019.00036)

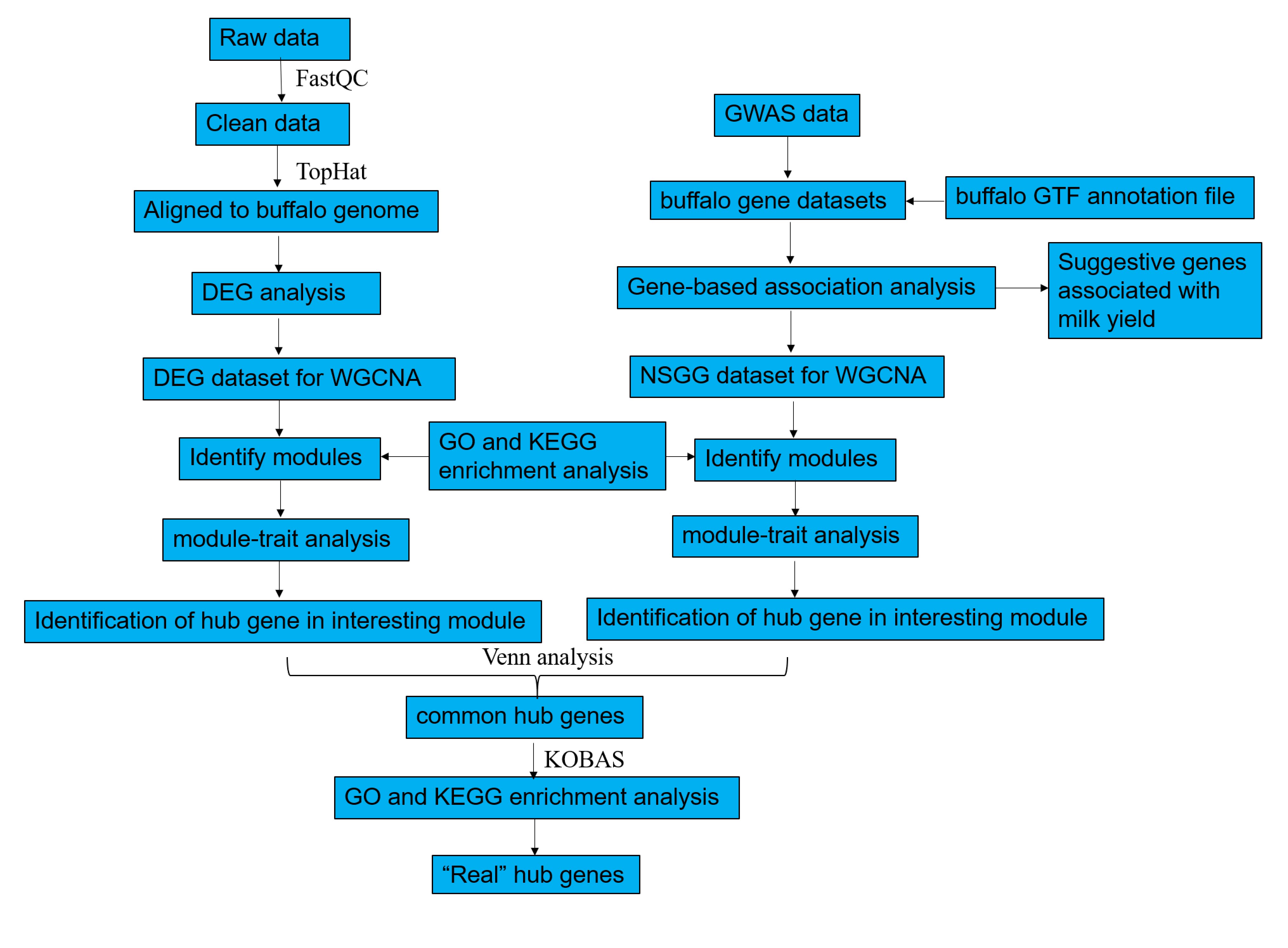

Supplement: FIGURE S1 — The main steps and bioinformatics used for data analysis in the present study. [file Image_1.JPEG]

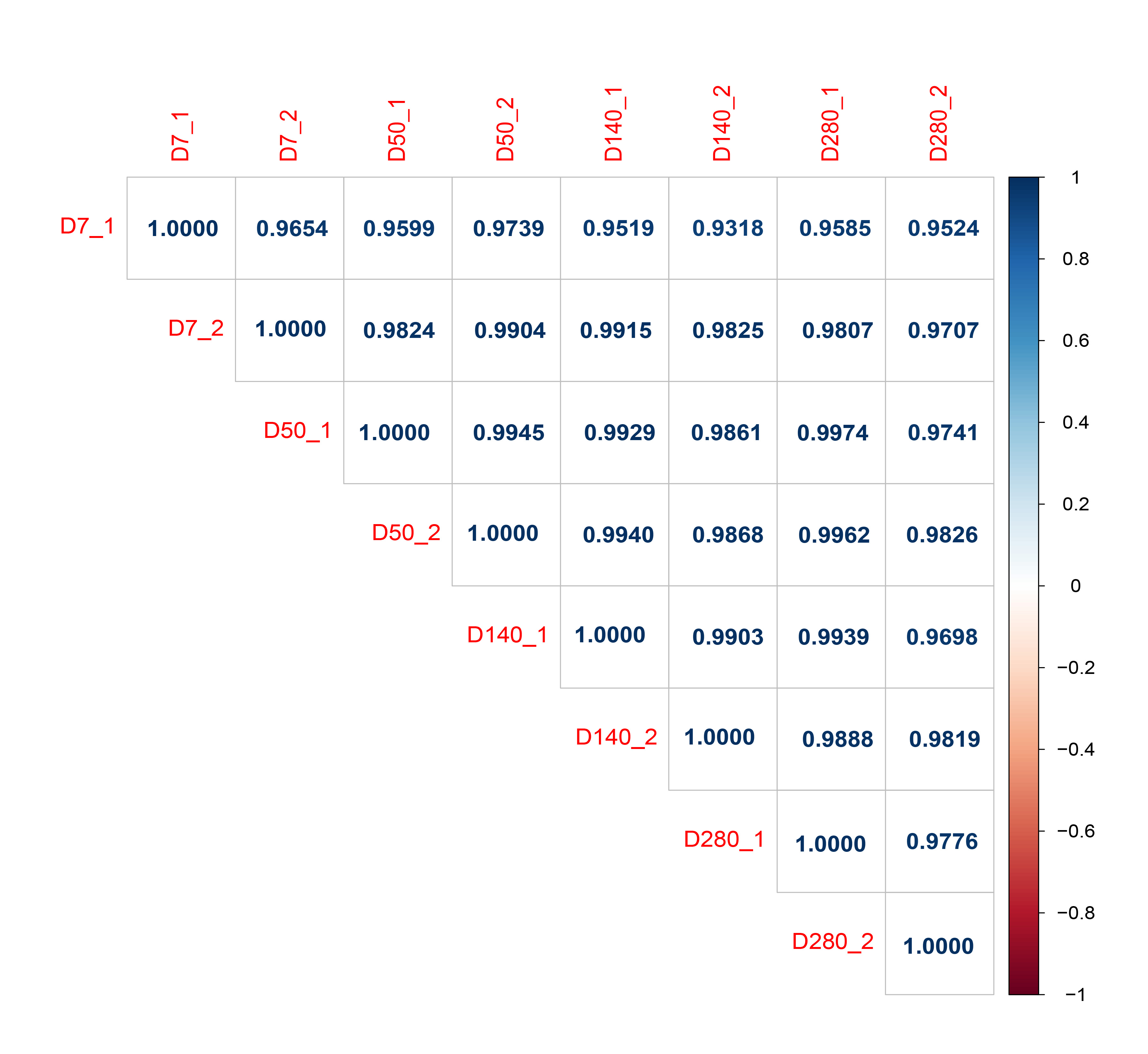

Supplement: FIGURE S2 — The PCA analysis of the studied samples in the present study. [file Image_2.JPEG]

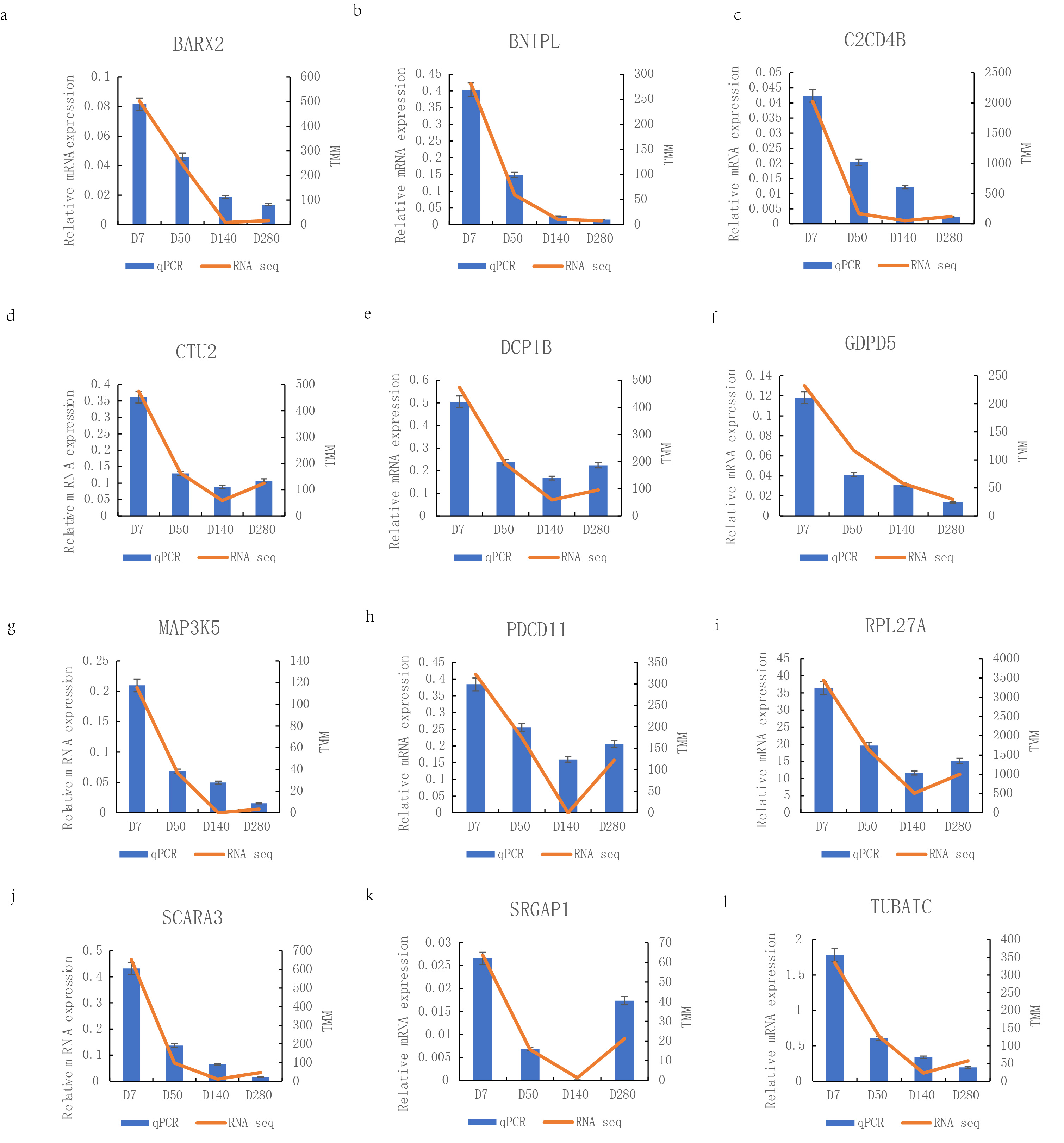

Supplement: FIGURE S3 — Validation of the expression of the 12 hub genes by qPCR. Gene expression levels were determined by qPCR and are presented as mean±SD values calculated by the 2ΔCt method. [file Image_3.JPEG]
